# Water treatment by H_2_O_2_ and/or UV affects carbon nanotube (CNT) properties and fate in water and tannic acid solution

**DOI:** 10.1007/s11356-015-5208-x

**Published:** 2015-08-26

**Authors:** Bożena Czech, Patryk Oleszczuk, Agnieszka Ewa Wiącek, Mariusz Barczak

**Affiliations:** Department of Environmental Chemistry, Faculty of Chemistry, Pl. Marii Curie-Skłodowskiej 3, 20-031 Lublin, Poland; Department of Interfacial Phenomena, Faculty of Chemistry, Pl. Marii Curie-Skłodowskiej 3, 20-031 Lublin, Poland; Department of Theoretical Chemistry, Faculty of Chemistry, Pl. Marii Curie-Skłodowskiej 3, 20-031 Lublin, Poland

**Keywords:** CNTs, Water treatment, Properties, Stability

## Abstract

The objective of the study was to estimate how water treatment (stimulation of real conditions) by H_2_O_2_ and/or UV affects carbon nanotube (CNT) properties and fate (stability/aggregation) in water and tannic acid solution. The processes studied had only a slight effect on S_BET_, porosity, and surface composition of CNTs. There was a change in the morphology of CNTs. After H_2_O_2_ and/or UV treatment, CNTs underwent shortening, opening up of their ends, and exfoliation. Treatment with H_2_O_2_ increased the content of oxygen in CNTs. A decrease was observed in the surface charge and in the mobility of CNTs, which caused an increase in their stability. UV irradiation of CNTs led to an increased incidence of defects that were manifested by both an increase of zeta potential and an increased mobility of CNT, whereas the presence of H_2_O_2_ during UV irradiation had only a slight effect on the parameters of the porous structure of nanotubes.

**Electronic supplementary material:**

The online version of this article (doi:10.1007/s11356-015-5208-x) contains supplementary material, which is available to authorized users.

## Introduction

The intensive development of technology, both in the area of the synthesis and of the study of materials, has contributed to the appearance and increasing production of new materials whose life cycle will end in the environment. Many of the compounds used currently are so-called engineered nanomaterials (ENM) (Nowack et al. 2013; Keller and Lazareva 2014). ENM, due to their specific physicochemical properties, are intensively used (Nowack et al. 2012).

At present, the production of NM is small, though there are no actual data but only estimates based on the production of products containing NM. The scale of production of carbon nanotubes (CNTs) is low (about 100 kg per year) (Nowack et al. 2012). The small-scale production of those materials means that the emission of CNTs to the environment should also be low. In spite of the potentially low current emission, CNTs may exert a negative effect on the environment, either directly or indirectly by affecting the fate of other contaminants.

In recent years, CNTs enjoy a notable interest, both on the industrial and the research scale (Newman et al. 2013). CNTs, as a result of direct release from materials during their use or storage, or indirectly, with wastes, biosolids, or treated wastewaters, may penetrate into the environment (Petersen et al. 2011; Nowack et al. 2013). It is predicted (Gottschalk and Nowack 2011; Keller and Lazareva 2014) that in wastewaters, CNTs will appear at concentrations from 0.01 to 0.05 μg/L, in sewage sludge from 0.05 to 0.1 mg/kg, and in aquatic environments, the concentration of CNTs is to attain levels from 0.003 to 0.02 ng/L. Those are small amounts, but even at such low concentrations, they will be able to affect organisms and exert an impact on the behavior of other contaminants in the environment (Oleszczuk et al. 2009; Gottschalk et al. 2009).

CNTs can undergo a variety of processes in the environment (Petersen et al. 2011). The subject of the behavior of nanotubes in the environment, in the context of their aggregation and deposition under the effect of environmental factors, has been researched extensively (Petosa et al. 2010; Qu et al. 2013). CNTs, like most contaminants, will penetrate into the water environment mainly via wastewater treatment plants, with treated wastewaters. However, there is a shortage of data concerning the fate of CNTs in wastewater treatment plants, especially in relation not so much to the efficiency of their removal as to the effect of the purification processes on nanoparticles (Mueller and Nowack 2010). Computer simulations indicate that the efficiency of removal of pristine CNTs during wastewater treatment in the presence of NOM should be >90 %. However, in a study on simulated coagulation and flocculation, the degree of elimination of CNTs was notably lower and amounted to 10–85 %, with relation to the concentration of CNTs in the wastewaters and to the kind and dose of the coagulating agent applied (Yi and Chen 2013).

The processes of wastewater and water treatment and purification commonly involve the use of oxidants such as O_3_, H_2_O_2_, UV, or Cl compounds, however in lower concentrations than used for the CNT oxidation. It is interesting though to study how the milder conditions that are observed during wastewater treatment will affect CNTs in treated water. Although CNTs are generally resistant to oxidation, it can take place on the ends of nanotubes. In the case of the walls of CNTs, oxidation can occur only at defect sites such as pentagon-heptagon pairs called Stone-Wales defects, sp^3^-hybridized defects, and vacancies in the nanotube lattice (Petersen et al. 2011). Though in the environment, the oxidation of CNTs is rather improbable and possible only in the form of photo-oxidation (Qu et al. 2013), such changes cannot be totally excluded as they do appear, e.g., in the course of sewage treatment. To date, research concerning CNT oxidation mainly focus on intentional oxidation of CNTs by strong acid or oxidizing agents in direction to obtain the new materials. In the present study, the stimulation of natural conditions of water treatment by H_2_O_2_/UV was applied to investigate how these treatments affect CNT properties and especially fate (aggregation/stability) in an aqueous and tannic acid solution. To our best knowledge, such studies have not yet been conducted.

The changes in the physicochemical properties of CNTs caused by the processes of wastewater and water treatment and purification will primarily affect the mobility of CNTs. It has been determined that the toxicity of CNTs is related to their surface properties and to the number of functional groups (Smith et al. 2009). Thus, there is the need to estimate what changes nanotubes undergo after being subjected to the processes of wastewater treatment or water purification.

In spite of the toxicity of NM themselves, there is also a high probability that, due to their particularly developed surface, they can adsorb—permanently or lightly—other contaminants and transport them within the environment. Contaminants adsorbed on CNTs can be (1) effectively retained or (2) released depending on the environmental conditions, and thus affect the toxicity of water (Schwyzer et al. 2012).

The objective of the proposed research was the estimation of the effect of UV irradiation (254 nm), H_2_O_2_, and UV with H_2_O_2_ on changes in the properties of multi-walled carbon nanotubes.

## Materials and methods

### Materials

**Fig. 1:**
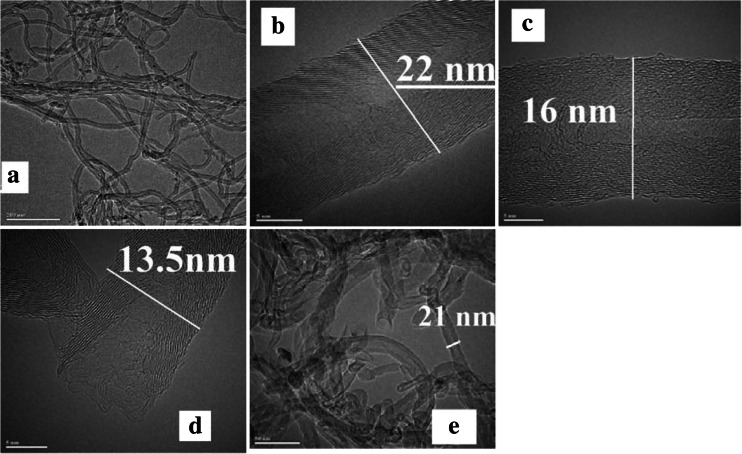
TEM images of studied CNTs. **a**, **b** CNT, **c** CNT-H_2_O_2_, **d** CNT-UV, **e** CNT-H_2_O_2_-UV

Multi-walled carbon nanotubes (CNTs) (Timesnano, China) were used in the present experiment. CNT were characterized by >95 % purity, specific surface area of 165.6 m^2^/g, 10–20-nm outer diameter, 10–30-μm length, and <1.5 wt.% of ash. TEM images are presented in Fig. 1a, b.

Hydrogen peroxide (H_2_O_2_) 30 % was purchased from POCH (Poland). Tannic acid (C_76_H_52_O_46_, TA) (Sigma-Aldrich, Poland) was used for the stabilization of nanotubes. Distilled water was used for the preparation of the water solutions.

### Water treatment

The studies were conducted in Heraeus reactor (0.75 L, Heraeus, Germany). The water solutions of 1 g/L CNT were treated by the following procedures: (1) 5 h of irradiation by UV mercury lamp (254 nm, 15 W, radiation flux 3 W, Heraeus, Germany), (2) the addition of 0.35 wt.% H_2_O_2_ (POCH, Poland), and (3) 5-h-long UV irradiation with 0.35 wt.% H_2_O_2_.

During the treatment, the reactor was isolated from the external source of light, and the mixture was magnetically stirred (120 rpm). After the treatment, the samples were filtered through paper filters (0.2 mm, CNTs recovery at least 97.8 %) and dried at 40 °C overnight and analyzed. According to the applied procedures, the nanotubes were labeled as follows: CNT-UV—depicted UV-irradiated nanotubes, CNT-H_2_O_2_—nanotubes treated by H_2_O_2_ and CNT-UV + H_2_O_2_ depicted UV and H_2_O_2_ treated carbon nanotubes.

### Carbon nanotube characteristic

The specific surface area of the CNTs was determined on the basis of low-temperature nitrogen adsorption-desorption method in Nova 1200e (Quantachrome Instruments, USA). *S*_*BET*_ were calculated using the standard BET method for nitrogen adsorption data, and pore size distribution was obtained from desorption branch of the isotherm according to BJH procedure. The elemental composition (C, H, N) of CNT was measured by CHN 2400 Analyzer (Perkin-Elmer, USA). Raman spectroscopy was applied using inVia Reflex (Renishaw, UK). The scans were collected for the range 150–3200 cm^−1^. The intensity was measured with the accuracy of 0.001 U. Mean diameter, electrophoretic mobility, and zeta potential were measured on Zetametr Zeta Plus Bi-Mass (Brookhaven Instruments Corporation, USA). The powder of CNT or modified CNT (5 mg) was dispersed in water (100 mL) by mechanical stirring at 10,000 rpm for 10 min (Heidolph Homogenizer) at room temperature. Water used in the experiments was demineralized and filtered with Milli-Q Plus 185 system (Milipore France, resistivity 18.2 MΩ cm). The electrophoretic mobility and mean diameters of suspension were determined by means of Brookhaven Zetameter via dynamic light scattering (DLS) (Grabowski 1983). Both parameters were measured for the same portion of the suspension soon after homogenization. Two measurement runs of diameter and the electrophoretic mobility were taken with five cycles in each run. The zeta potential was calculated from the mobility data using the Helmholtz-Smoluchowski equation, and the reproducibility of the results was better than 95 %.

TEM studies were conducted on a Titan G2 60–300 (FEI) guaranteeing the work of field beam accelerating voltage in the range of 60–300 kV and a resolution of less than 0.1 nm. For the studies, the sonicated CNT mixtures in methanol were placed on the net. The scans were collected in broad zoom spectrum and enabled to observe nanotubes in the range of 5–200 nm.

### Stability of carbon nanotubes

The stability and aggregation of CNTs in water or tannic acid (TA) (Sigma-Aldrich, Poland) solution were investigated at the concentration of 2 mg CNTs/L. The concentration of TA was at the level of 30 mg/L. In previous research (Lin and Xing 2008a; Lin et al. 2009), the stabilizing effect of TA was observed for the concentration starting from 20 mg/L TA that is representative for the dissolved organic matter in environment. A stable suspension was obtained after sonication (30 min) and was stored in dark parafilm closed bottles at room temperature until use. Sonicated samples were analyzed by UV-Vis spectrophotometry (Shimadzu UV-2700, USA) cutting edge following route. The first sample was analyzed after 30 min after mixing; the next measure of constantly stirred (120 rpm) nanotubes was conducted after 4 and 24 h. After 24 h of mixing, the samples were left for settling. The first point of settling kinetics was measured after 4 h of settling, and next were analyzed after 24, 96, and 168 h of settling. The stability of nanotubes was determined using absorbance measurement at 800 nm that was recognized as characteristic for carbon nanotubes (Schwyzer et al. 2011). Each experiment was run in triplicate.

## Results and discussion

### Specific surface area

Table 1 and Fig. S1 (supplementary material) present the results of measurements of nitrogen adsorption and desorption on CNTs subjected to simulated processes of purification. All the isotherms can be classified as type II according to IUPAC classification (Rouquerol et al. 1999). The isotherm of pristine sample presents a small initial increase in adsorption due to the presence of some fraction of micropores. A slow increase in the amount of adsorbed nitrogen is observed at the intermediate p/p_o_ (0 < p/p_o_ < 0.7), indicating that the adsorption of nitrogen on the external surfaces of the nanotubes slow increase occurs via layer-by-layer adsorption. A hysteresis loop (as the result capillary condensation), associated with high increase of the nitrogen uptake, is located at very high relative pressures (p/p_o_∼0.8–0.95). This testifies to the presence of mesoporosity coming from (i) voids between entangled nanotubes (nanotubes interact via intermolecular forces forming entangled aggregates (Błachnio et al. 2008) and (ii) from the central canals of the open tubes.

**Table 1:** The characteristic of multi-walled carbon nanotubes before (CNTs) and after treatment with H_2_O_2_ (CNT-H_2_O_2_), UV (CNT–UV) and UV, and H_2_O_2_ (CNT–UV+ H_2_O_2_)

CNTs	BET surface (m^2^/g)	Pore volume (cm^3^/g)	Pore radius (Å)	ID/IG (-)	Bulk C (%)	Bulk H (%)	Bulk N (%)	Bulk O (%)	O/C (-)	H/C (-)	(N + O)/C (-)
CNT	165.6	0.90	109.0	1.23	96.26	0.14	0.49	3.11	0.032	0.0015	0.037
CNT- H_2_O_2_	162.5	1.28	157.7	1.10	96.13	0.00	0.49	3.39	0.035	–	0.040
CNT -UV	166.3	1.29	155.0	1.48	96.48	0.00	0.49	3.04	0.032	–	0.037
CNT – UV+ H_2_O_2_	169.6	0.94	110.9	1.28	96.17	0.00	0.43	3.41	0.035	–	0.040

The oxidized nanotubes also show a type II isotherm with similar features to the isotherm of the pristine sample, although the hysteresis loops are noticeably wider and better pronounced what can indicate a partial uncapping of the nanotubes as a result of the oxidation treatment. However, this oxidation is not so robust as in the case of wet chemical oxidation; such wet treatment causes severe changes in the mesopore structure who are reflected in the shape of the hysteresis loop (Vicente et al. 2011).

The data acquired suggest different effects of H_2_O_2_ and UV (Fig. 1, Table 1). The increase in the value of total surface area after the application of UV indicates that under the effect of irradiation, there may take place cracking of the outer walls of CNTs and, most likely, the appearance of numerous defects as the result of action of radicals generated under UV irradiation, whereas the only slightly changed parameters of *S*_*BET*_, but drastically increased values of the other parameters, indicate that H_2_O_2_ may penetrate into the deeper layers of nanotubes, causing also an increase in pore volume. There are two explanations for this phenomenon. First, the pores may have become elongated, which could be related with “expansion” of pores in their broadest points, most likely at the ends of open-ended nanotubes. On the other hand, it is assumed (Bennett et al. 2013) that the distance between nanotubes is responsible for their porosity. Therefore, cracking of surface layers and introduction of functional groups may result in processes of electrostatic repulsion between nanotubes, which leads to an increase of the volume of space between them. When H_2_O_2_ is applied in conjunction with UV irradiation, increased porosity parameters were noted. In this case, H_2_O_2_ could be another source of radicals that were formed after UV irradiation.

### Elemental composition of CNTs

The application of oxidants results also in changes in the elemental composition of CNTs (Table 1). The content of *C%* is at the same level irrespective of the method applied. This indicates that both UV radiation and H_2_O_2_ are unable to cause a drastic change in the composition of nanotubes. The absence of significant changes is due to the fact that CNTs are fairly resistant to degradation and transformations, which is supported by earlier research (Qu et al. 2013).

The key parameter is the change in the amount of oxygen which determines the sorptive abilities of nanotubes. Potential oxidation processes can take place on the outer surface of nanotubes (Kotchey et al. 2013), which can be observed in the slightly increased level of oxygen after the application of H_2_O_2_ and UV/H_2_O_2_, whereas UV radiation applied alone causes a decrease in the level of oxygen. A reduction of the level of oxygen for CNTs treated with UV indicates an increase of ester or –COH bonds at the cost of other oxygen bonds. Lower content of oxygen after treating of CNTs with UV may suggest also that UV generates processes of mineralization of nanotubes, most likely as a result of the effect of ·OH radicals generated under the effect of UV (Qu et al. 2013). A reduction of *H*% may indicate a change in the character of *C–C* bonds and an increase of aromaticity of nanotubes. This is confirmed by decreased value of the ratio *H/C*, as a measure of aromaticity of carbonaceous materials, indicating that after the treatment, nanotubes have a more aromatic and carbonized character. Additional information on the character of surface of nanotubes is provided by the parameter (N + O) / C − polarity index. Its increase for nanotubes treated with H_2_O_2_ and UV/H_2_O_2_ means an increase of polarity.

### Size of CNTs

H_2_O_2_ exerts an impact on the size of aggregates of CNTs, causing them to nearly double (Table 2). Worth noting is the fact that in spite of the large mean size of particles, the dominant fraction is that of aggregates with size under 1 μm. UV irradiation induces a slight reduction in the aggregate size. It also needs to be emphasized that both with an addition of H_2_O_2_ alone and with UV irradiation in the presence of H_2_O_2_, there appear, though in a small amount, concentrations of nanotubes with the same hydrodynamic diameter −∼9 μm. Also, all the nanotubes are characterized by multimodal size distribution, notably increased for systems treated with H_2_O_2_. The increase of stability, usually manifested by a decrease in the size of aggregates, e.g., CNT-UV, may be a result of the presence of negative charge on the outer surface of nanotubes, originating from negatively charged groups or products after the process of oxidation (Li et al. 2013a), although the steric stabilization (Schwyzer et al. 2013), characteristic for systems with polymers, can also be taken into account as nanotubes constituting here long hydrophobic chains.

**Table 2:** Mean aggregates diameter and zeta potential of multi-walled carbon nanotubes studied

CNTs	Mean aggregates diameter (nm)	Size distribution (nm)	Dispersity (−)	Zeta potential (mV)	Mobility (m^2^/s*V)
CNT	2968.7 ± 240.9	125.1 (87.6 %) 3146.6 (1.26 %)	0.306	9.54 ± 5.12	0.68 ± 0.36
CNT-H_2_O_2_	6687.5 ± 3734.4	449.3 (87.7 %) 8985.4 (≤1 %)	0.507	2.71 ± 10.01	0.19 ± 0.71
CNT-UV	2630.1 ± 164.9	781.57 (90.3 %) 9030.6 (≤1 %)	0.365	12.91 ± 2.03	0.92 ± 0.14
CNT-H_2_O_2_-UV	9154.2 ± 3644.5	556.5 (83.9 %) 8985.4 (≤1 %)	0.378	−1.36 ± 6.8	−0.1 ± 0.49

### TEM

The results of TEM analyses are presented in Fig. 1. Nanotubes not treated with H_2_O_2_/UV (Fig. 1a, b) were characterized by relatively long and straight chains with closed ends. Generally, the application of UV irradiation or the presence of H_2_O_2_ affect the structure and morphology of nanotubes. In both cases, CNTs get shortened, their ends open up, and the walls undergo exfoliation (Fig. 1c, d), while the distance between the preserved walls remains unchanged at 0.2 nm. The number of walls of CNTs becomes reduced from 35 to 21–25 after the application of UV (Fig. 1d) and to 11 after the application of H_2_O_2_ or UV in the presence of H_2_O_2_ (Fig. 1e). H_2_O_2_ induces significant changes in the character of the surface, structures crack, and “protruding” chains appear (Fig. 1c).

Following UV and H_2_O_2_ treatment, the structure of CNTs resembles beads, and wall cracking and “wound healing” are visible (Fig. 1e). The observed shortening of nanotubes or exfoliation may result from ·OH reaction, responsible for the mineralization of nanotubes (Qu et al. 2013). The TEM analyses are mutually complementary with data obtained from Raman spectroscopy (discussed in a further section of the paper). It should be emphasized that the changes observed so far in the surface area or length of nanotubes, the presence of amorphous carbon, degree of dispersion or number of walls have an effect on the toxic and cytotoxic properties of CNTs (Upadhyayula et al. 2009). These changes decrease aggregation ability of CNTs and increase affinity of CNTs to cell membranes (Muller et al. 2009).

### Stability and zeta potential

For the determination of surface charge densities, dispersibility and mobility of carbon nanotube measurements of zeta potential were used. Dynamic light scattering (DLS) is the powerful method for measurements of nanoparticle system size and provides information on the size distribution of the dispersed particles by analysis of the autocorrelation function of the laser light scattered by the particles undergoing Brownian motion. Zeta potential is useful indicator of particle surface charge to predict the stability of systems during the storage and in the different medium. At this time, it is stated that the zeta potentials above 20–25 mV are required for total electrostatic stabilization. This rule cannot be precisely applied for the systems which contain steric stabilizers, e.g., polymers. In such systems, steric stabilization should be taken into account.

The processes studied affected the electric properties and the mobility of CNTs. The increase of the ζ-potential indicates that electrostatic repulsion of the functional groups imparted by the dispersing agent/via covalent modification is sufficient to overcome the attractive van der Waals interactions of the CNTs (Kotchey et al. 2013). Generally, the nanotubes, both in the pristine form and after treatment with H_2_O_2_ and/or UV, have relatively small surface charge (Table 2). H_2_O_2_ causes a decrease in the value of the zeta potential by nearly 7 mV, whereas UV causes an increase of the positive surface charge of nanotubes by 3.4 mV. Increase of the value of zeta potential of nanotubes informs that there is a decrease of electrostatic repulsion, and thus that aggregation and sedimentation are faster, which has been additionally confirmed by further analyses of stability. Increase in the value of the ζ potential results in greater electrostatic repulsion of the functional groups introduced via the covalent modification or the appearance of dispersing factors, which leads to the overcoming of the van der Waals attraction forces, and to the orientation of negatively charged carboxyl groups of nanotubes in the direction of positively charged residue (Kotchey et al. 2013).

The greatest decrease of ζ-potential was observed for nanotubes treated with H_2_O_2_ and UV simultaneously. Decrease of zeta potential due to treatment represents an increase of the negative charge on the surface from the introduction of oxygen, predominately present as carbonyl moieties, on the surface into sp^3^ carbon (Ellis et al. 2007; Lin et al. 2009; Li et al. 2013a), which is supported by the results concerning the level of *O%* in nanotubes subjected to water purification processes with UV. Although the results obtained for H_2_O_2_ do not conform to this relationship, that relation cannot be excluded on the grounds of the large measurement error involved.

**Fig. 2:**
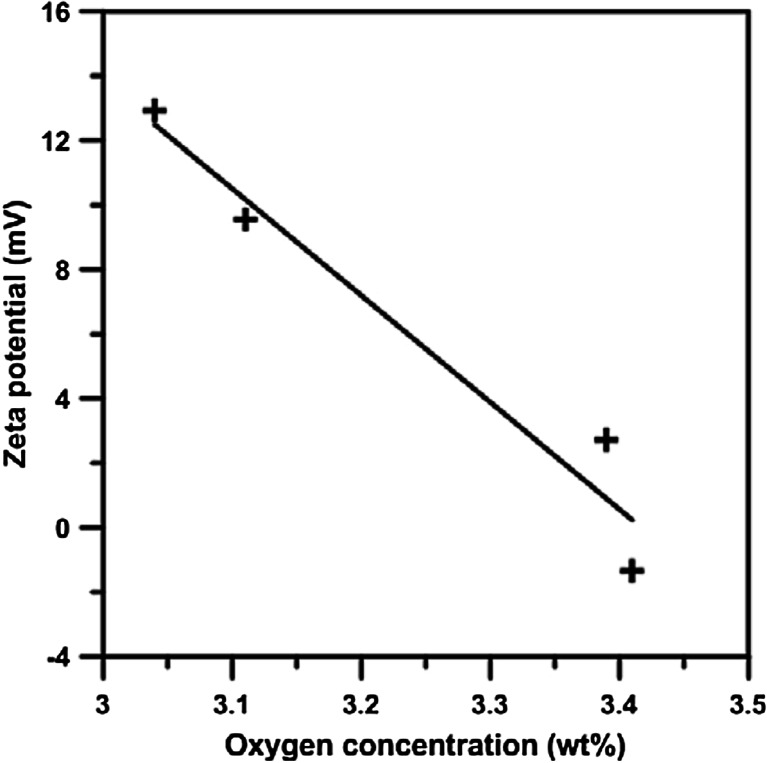
The relationship between zeta potential and oxygen content of studied CNT

It is worth noting that the mobility of CNTs treated with H_2_O_2_ decreases. Among the nanotubes, the greatest mobility, increased by 35 %, was displayed by CNT-UV. The increased mobility can result from the fact of generation of charge carriers with a rather high efficiency on the surface of nanotubes by UV (Qu et al. 2013). Therefore, the key parameter affecting the value of the surface charge is the content of oxygen in nanotubes, whereas the reactivity of various oxygen groups is not identical. Literature data indicate that nanotubes with a low content of oxygen are usually characterized by a low stability in water suspension, the stability being directly proportional to oxygen content (Li et al. 2013a), which was confirmed in further studies on stability. A distinct correlation between zeta potential and oxygen content of CNTs was observed in this study (Fig. 2). Oxygen content in CNTs may have an indirect effect on the adsorption abilities of nanotubes (Lin and Xing 2008b; Li et al. 2013a). It should, therefore, be expected that apolar compounds will be more easily adsorbed on pristine nanotubes and polar ones on CNTs after treatment with H_2_O_2_ and/or UV.

### Raman spectroscopy

**Fig. 3:**
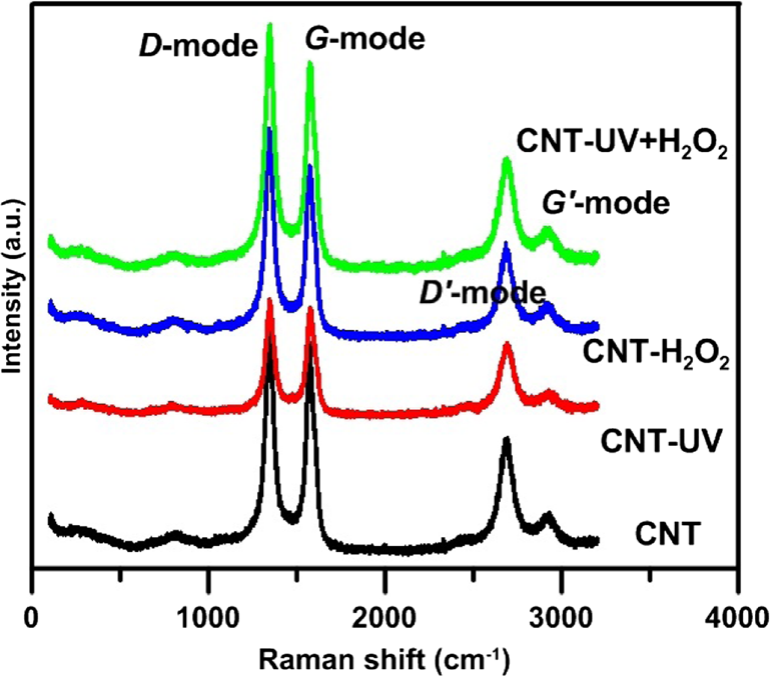
Raman spectrum of studied CNTs before (CNT) and after treatment with H_2_O_2_ and/or UV

The surface of CNTs after treatment with H_2_O_2_ and/or UV was characterized by means of Raman spectroscopy. The spectrum presented in Fig. 3 shows 4 peaks characteristic for carbon nanotubes, with an almost identical intensity for all the systems studied: (i) distinct peak ∼1565 cm^−1^ (*G band*) related with the appearance of well graphitized CNTs, including perfect interaction of intramolecular vibration between *C* atoms; (ii) peak at ∼1345 cm^−1^—the *D band* refering to *C–C* stretching vibrations disordered graphite (heptagon and pentagon in graphite), defective structure in the tube wall and/or an existence of a-C (Ellis et al. 2007; Li et al. 2011), and also two other peaks. The first at nearly ∼2689 cm^−1^ (*D*′) assigned to the first overtone of band *D* and the second *G′* at ∼2917 cm^−1^ which is a combination of *D* and *G* modes.

Taking into account the intensity of occurrence of the particular peaks, decisively, the lowest is that for nanotubes treated with H_2_O_2_. Although the D-band is weaker, it still relates to intrinsic impurities (defects disordered and amorphous carbon). UV causes a slight decrease in the intensity of the peaks in the Raman spectrum, and all the factors combined (H_2_O_2_ and UV) cause a slight increase in the intensity of *D-* and *G-mode*. In the Raman spectrum, the intensity of radiation exiting the sample is a linear function of concentration and layer thickness, and thus, it supports the earlier TEM analyses, different numbers of concentric layers, and different outer diameters.

As the *D mode* is related with the occurrence of defects or amorphous carbon and *G-band*—with well-ordered sp^2^ carbon on the graphitic sidewall—the parameter that describes the occurrence of defects is the ratio of intensity of the two peaks, *ID/IG*. The lower the value of *ID/IG*, the more graphitized the nanotubes (Ellis et al. 2007).

Values of *ID/IG* ratio are presented in Table 1. The presence of H_2_O_2_ causes a decrease (by 10 %) of the value of that parameter, while UV radiation causes a significant increase (by 20 %). An absence of significant changes in the values of ID/IG was observed for H_2_O_2_/UV. Decrease in the value of *ID/IG* can be attributed to the exfoliation of highly oxidized graphitic lattice from the nanotube surface or its degradation, which is known to lower the *D-band* intensity as the inner graphitic sidewall is exposed (Qu et al. 2013). Increase of *ID/IG* suggests an increased number of defects in nanotube walls, but it can also result from domination of sp^3^-hybridization over sp^2^-hybridization of nanotubes (Shamsudin et al. 2012). The areas of occurrence of defects on the walls and edged of nanotubes can become biodispersible and biocompatible (Kotchey et al. 2013). Summarizing, H_2_O_2_ is a factor permitting easier exfoliation of external walls of nanotubes, which would confirm the results of studies conducted with methods applied before. UV is responsible for the generation of ·OH radicals and for the appearance of defects, whereas, when both factors are applied jointly, in spite of the photolysis of H_2_O_2_ under the effect of UV, the primary mechanism of the changes is increased generation of defects in nanotubes.

### Stability of CNTs

The most important parameter characterizing CNTs is their ability to agglomerate, which affects sedimentation and mobility (Quik et al. 2014) and biodavailability in the environment (Li et al. 2013b) and, in consequence, their toxicity (Klaine et al. 2012). The extent of aggregation is related with the numbers of functional groups on the surface of nanotubes, mainly oxygen ones. The larger the number of functional groups, the greater the critical coagulation concentrations.

**Fig. 4:**
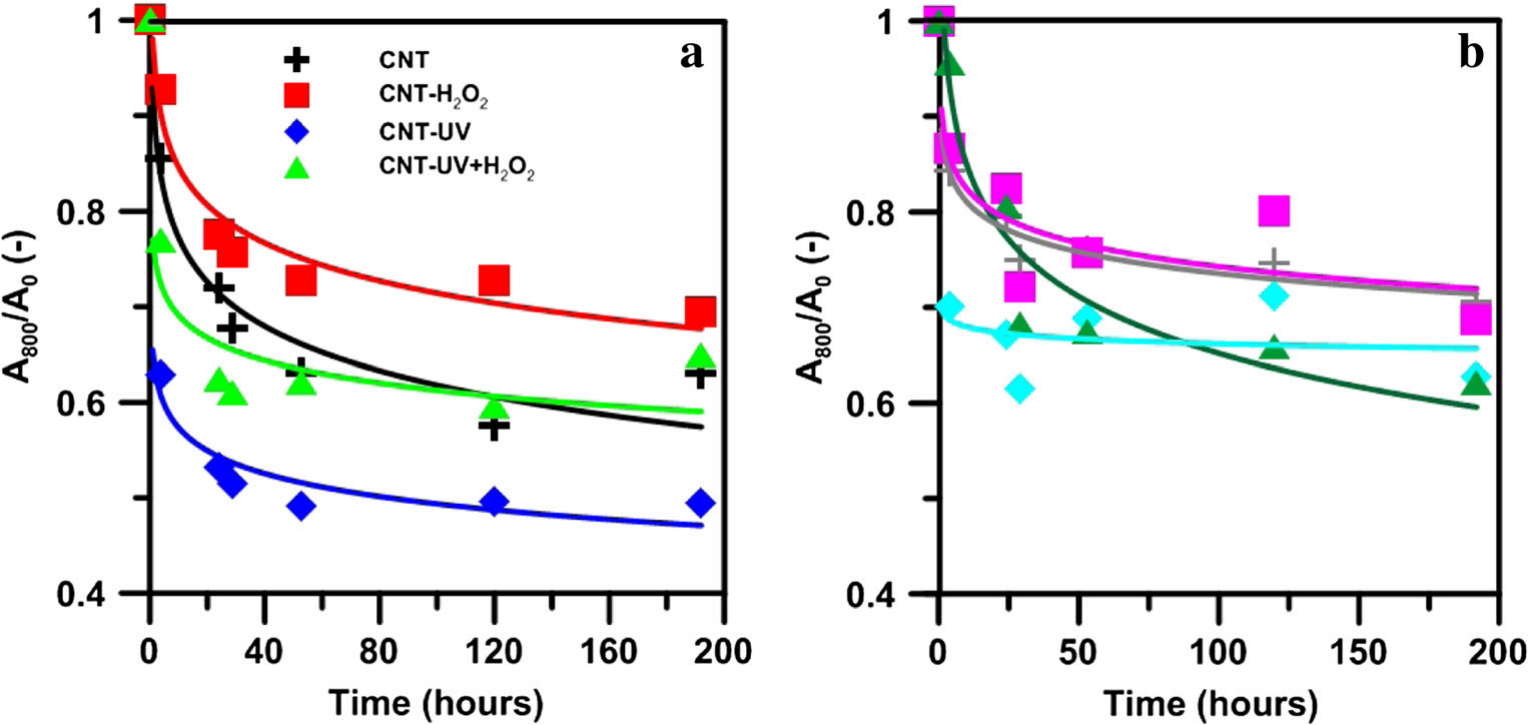
The stability of CNTs in **a** water and **b** tannic acid solution

Figure 4 presents the stability of the studied CNTs prior to and after the process of their treatment with UV and/or H_2_O_2_. Basically, CNTs display a tendency to aggregate in bundles, ropes, and networks or lattices under the effect of van der Waals forces (Kotchey et al. 2013). The effect of application of water purification processes by means of H_2_O_2_ or UV or both of the factors in conjunction on the sedimentation ability of nanotubes is not identical. Generally, the nanotubes agglomerated fairly rapidly, which caused their sedimentation. Usually, the nanotubes attained stability after 24–48 h. Afterward, a gradual decrease of stability could be observed, but it was not as significant as during the initial 24/48 h. These observations are in conformance with the data presented by Schwyzer et al. (2011) who also found that after 24 h, distinct sedimentation of CNTs took place, until the appearance of a plateau that persisted for at least 10 days.

H_2_O_2_ has a stabilizing effect on CNTs, while UV causes easier aggregation and sedimentation of CNTs. In the case of treating nanotubes with UV and H_2_O_2_ simultaneously, their effects neutralize each other, and the agglomerates, though initially, they sediment faster than pristine CNT, the stabilization attained ultimately is at a similar level.

The addition of TA, aimed at the simulation of the presence of dissolved organic matter (Lin et al. 2009), always caused an increase of stability compared to water solution of carbon nanotubes. TA increased not only the amount of suspended particles, but also the time of reaching an equilibrium that in many cases was attained as soon as after 4 h of sedimentation. The effect of TA was the most pronounced in the case of CNT-UV (Fig. 4b), and the least for CNTs treated with H_2_O_2_. The stabilizing effect of TA results from interactions between TA and CNTs. Monolayer adsorption of TA via *π*-*π* interactions of the aromatic rings of TA and nanotubes, as well as other polar interactions (e.g., hydrogen bonds with the dissolved TA), result in increased steric repulsion between individual CNTs (Lin and Xing 2008a).

The formation of dispersed stable suspensions of CNTs (individual nanotubes in small bundles) is achieved through (1) the formation of defects as a result of chemical oxidation by a strong oxidant and (2) covalent chemistry performed on sidewall (Kotchey et al. 2013). In this case, the increased stability is most probably due to increased number of defects and to changes in the outer walls of nanotubes (Kotchey et al. 2013). This information has an extremely important environmental aspect. Increased number of defects was a result in a potential increase of biocompatibility and bioavailability, and thus toxicity, but also in easier degradation of nanotubes, e.g., under the effect of soil enzymes (Lin et al. 2009; Kotchey et al. 2013). The degree of agglomeration is also a factor determining toxicity, as semidispersible and partially hydrophobic nanotubes are more easily for organisms, e.g., bacteria, than weakly or totally soluble CNTs (Upadhyayula et al. 2009).

## Conclusions

H_2_O_2_ causes exfoliation of outer walls of CNTs, shortening, and opening up of their ends. The slight changes of S_BET_ and porosity were observed. H_2_O_2_ induces also changes in composition of CNTs, increasing their content of *O*%, reducing surface charge and mobility, and increasing the stability.

UV leads to the generation of radicals and to increased incidence of defects manifested by both increased zeta potential and mobility. UV causes also the cracking of outer walls of nanotubes and their exfoliation, but to a lesser degree than H_2_O_2_. However, the wall cracking is deep enough to cause an increase in the value of *S*_*BE*T_ and porosity.

The addition of H_2_O_2_ during UV irradiation has only a slight effect on the porosity of nanotube structure. Nevertheless, it induces qualitative changes, distinctly twisting nanotubes and opening up their ends. The TEM images show numerous cracks of the walls and reduction of the number of layers. The changes are caused by the formation of functional groups on the surface, which is reflected in increased O content, but also in the negatively charged surface. The value of the zeta potential and mobility is also reduced. In spite of the application of H_2_O_2_ and UV, and their different effects, when the two factors are applied jointly, the dominant factor modifying CNTs is UV.

## Electronic supplementary material

ESM 1 (DOC 115 kb)
