# Supplementary material for: Water treatment by H2O2 and/or UV affects carbon nanotube (CNT) properties and fate in water and tannic acid solution
Source: Environ Sci Pollut Res Int. 2015 Aug 26;22:20198–206. doi: 10.1007/s11356-015-5208-x (PMC4679106; doi:10.1007/s11356-015-5208-x)
Supplement: Supplementary file 1 — (DOC 115 kb) [file 11356_2015_5208_MOESM1_ESM.doc]

# WATER TREATMENT BY H2O2 AND/OR UV AFFECTS CARBON NANOTUBES (CNTs) PROPERTIES AND FATE IN WATER AND TANNIC ACID SOLUTION

Bożena Czech^1^, Patryk Oleszczuk^1*^, Agnieszka Wiącek^2^, Mariusz Barczak^3^

^1^Department of Environmental Chemistry, Faculty of Chemistry, 3 Maria Curie-Skłodowska Square 3, 20-031 Lublin, Poland

^2^Department of Interfacial Phenomena, Faculty of Chemistry, 3 Maria Curie-Skłodowska Square 3, 20-031 Lublin, Poland

^3^Department of Theoretical Chemistry, Faculty of Chemistry, 3 Maria Curie-Skłodowska Square 3, 20-031 Lublin, Poland

*corresponding author: [patryk.oleszczuk@umcs.lublin.pl](mailto:patryk.oleszczuk@umcs.lublin.pl), tel. +48 81 537 55 15, fax: +48 81 537 55 65

Journal: Environmental Science and Pollution Research

Number of pages: 2 (including this page)

Number of figures: 1


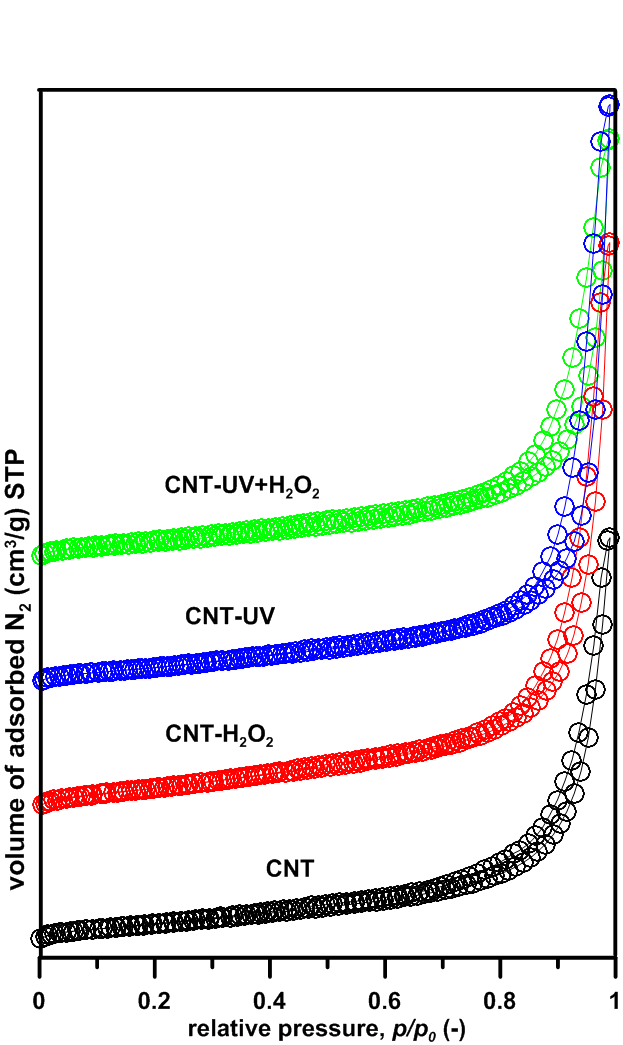


Figure S1. N_2_ adsorption-desorption isotherms of studied CNTs
